# Supplementary material for: N-Glycosylation Patterns across the Age-Related Macular Degeneration Spectrum
Source: Molecules. 2022 Mar 8;27(6):1774. doi: 10.3390/molecules27061774 (PMC8949900; doi:10.3390/molecules27061774)
Supplement: Supplementary file 1 [file molecules-27-01774-s001.zip › molecules-1555543-supplementary.pdf]

Table S1. Glycan measurements across the analysed groups, with pari-wise significance testing

Glycan peak	Bilateral AMD (B)	Unilateral AMD (U)	Early-onset drusen (e)	Controls (c)	P (F)	Significance, pair-wise comparison
Plasma glycans						
GP1	0.17±0.07 (0.10-0.37)	0.18±0.06 (0.08-0.27)	0.12±0.06 (0.05-0.25)	0.17±0.12 (0.03-2.11)	0.438 (0.91)	
GP2	4.13±1.23 (1.90-7.14)	4.35±1.25 (3.01-6.37)	2.75±0.81 (1.59-4.66)	3.74±1.39 (1.06-12.65)	0.009 (3.84)	ce 0.033; eU 0.036; eB 0.013
GP3	2.14±0.37 (1.27-2.72)	2.34±0.33 (1.87-2.97)	2.00±0.30 (1.58-2.48)	2.09±0.46 (1.07-5.97)	0.318 (1.18)	
GP4	5.17±1.26 (2.87-7.22)	5.89±0.91 (4.62-7.82)	5.43±1.03 (3.64-7.29)	5.4±1.25 (1.99-11.07)	0.522 (0.75)	
GP5	2.34±0.35 (1.60-2.97)	2.29±0.30 (1.93-2.83)	2.53±0.44 (2.04-3.78)	2.42±0.43 (1.35-4.09)	0.459 (0.86)	
GP6	3.44±0.74 (2.13-4.91)	3.78±0.51 (3.27-4.85)	4.28±1.00 (2.82-6.41)	3.77±0.91 (1.49-7.39)	0.043 (2.72)	eB 0.026
GP7	11.97±4.08 (8.32-26.34)	10.01±1.1 (8.17-11.42)	10.00±1.26 (8.14-13.15)	10.75±2.42 (6.53-29.18)	0.038 (2.82)	
GP8	8.8±1.19 (6.06-11.01)	9.42±0.87 (7.86-10.39)	10.43±2.10 (7.45-16.51)	9.41±1.55 (5.74-14.93)	0.016 (3.45)	eB 0.008
GP9	37.78±2.68 (31.95-42.03)	38.41±1.96 (34.71-40.52)	38.02±2.52 (32.94-40.91)	37.93±3.09 (22.64-50.5)	0.962 (0.10)	
GP10	7.26±1.5 (4.18-10.25)	6.74±1.25 (5.26-8.66)	7.53±1.24 (5.22-10.12)	7.32±1.59 (3.39-15.54)	0.676 (0.51)	
GP11	2.10±0.66 (1.15-4.21)	2.06±0.69 (1.24-3.19)	1.81±0.37 (1.39-2.71)	2.05±0.69 (0.81-5.7)	0.584 (0.65)	
GP12	1.61±0.41 (0.95-2.52)	1.71±0.49 (1.36-2.96)	1.87±0.72 (1.16-3.20)	1.8±0.53 (0.65-3.72)	0.306 (1.21)	
GP13	4.62±1.45 (1.82-7.47)	5.10±0.86 (3.74-6.29)	5.40±1.37 (3.28-7.97)	5.28±1.47 (0.59-11.18)	0.154 (1.75)	
GP14	7.19±1.72 (4.27-10.68)	6.40±0.89 (5.25-7.99)	6.34±1.28 (4.43-9.68)	6.46±1.44 (2.51-12.54)	0.094 (2.14)	
GP15	0.44±0.18 (0.10-0.84)	0.53±0.13 (0.39-0.79)	0.55±0.21 (0.21-0.89)	0.53±0.19 (0.06-1.51)	0.141 (1.82)	
GP16	0.83±0.40 (0.31-2.03)	0.80±0.34 (0.50-1.59)	0.95±0.34 (0.31-1.64)	0.87±0.31 (0.11-2.53)	0.656 (0.54)	
DG1	0.17±0.07 (0.10-0.37)	0.18±0.06 (0.08-0.27)	0.12±0.06 (0.05-0.25)	0.17±0.12 (0.03-2.11)	0.438 (0.91)	
DG2	4.13±1.23 (1.90-7.14)	4.35±1.25 (3.01-6.37)	2.75±0.81 (1.59-4.66)	3.74±1.39 (1.06-12.65)	0.009 (3.84)	ce 0.033; eU 0.036; eB 0.013
DG3	2.14±0.37 (1.27-2.72)	2.34±0.33 (1.87-2.97)	2.00±0.30 (1.58-2.48)	2.09±0.46 (1.07-5.97)	0.318 (1.18)	
DG4	5.17±1.26 (2.87-7.22)	5.89±0.91 (4.62-7.82)	5.43±1.03 (3.64-7.29)	5.4±1.25 (1.99-11.07)	0.522 (0.75)	
DG5	2.34±0.35 (1.60-2.97)	2.29±0.03 (1.93-2.83)	2.53±0.44 (2.04-3.78)	2.42±0.43 (1.35-4.09)	0.459 (0.86)	
DG6	3.44±0.74 (2.13-4.91)	3.78±0.51 (3.27-4.85)	4.28±1.00 (2.82-6.41)	3.77±0.91 (1.49-7.39)	0.043 (2.72)	eB 0.026
DG7	11.97±4.08 (8.32-26.34)	10.01±1.1 (8.17-11.42)	10.00±1.26 (8.14-13.15)	10.75±2.42 (6.53-29.18)	0.038 (2.82)	
DG8	8.80±1.19 (6.06-11.01)	9.42±0.87 (7.86-10.39)	10.43±2.1 (7.45-16.51)	9.41±1.55 (5.74-14.93)	0.016 (3.45)	eB 0.008
DG9	33.67±10.77 (5.22-42.03)	34.32±11.63 (3.68-40.45)	33.59±12.22 (3.66-40.91)	33.46±11.68 (0.42-50.5)	0.996 (0.02)	
DG10	2.82±2.75 (0.82-8.92)	3.84±3.45 (0.87-8.66)	4.28±3.54 (1.03-10.12)	3.75±3.29 (0.54-13.83)	0.496 (0.80)	
DG11	2.25±0.60 (1.05-3.68)	2.41±0.42 (1.9-3.07)	2.20±0.47 (1.52-3.04)	2.36±0.57 (0.9-5.03)	0.496 (0.80)	
DG12	0.93±0.46 (0.42-2.29)	0.78±0.2 (0.36-1.06)	0.73±0.25 (0.39-1.14)	0.75±0.30 (0.1-2.46)	0.029 (3.03)	cB 0.017
DG13	0.95±0.57 (0.32-2.30)	0.7±0.19 (0.34-0.97)	0.60±0.24 (0.28-1.15)	0.68±0.28 (0.18-1.95)	<0.001 (7.75)	cB <0.001; eB 0.001
Monosialo	24.69±4.13 (20.04-37.71)	22.9±1.62 (20.5-25.53)	23.72±3.2 (19.47-31.43)	23.83±3.44 (11.88-42.36)	0.512 (0.77)	
Disialo	57.38±2.65 (51.33-61.62)	59.06±0.92 (58.02-60.88)	58.18±2.03 (53.96-61.92)	58.06±2.32 (47.4-67.67)	0.269 (1.31)	
Trisialo	14.88±2.48 (8.26-19.00)	15.21±1.6 (11.88-16.68)	14.8±1.7 (10.98-17.51)	14.84±2.08 (6.06-22.27)	0.962 (0.10)	

Tetrasialo	3.05±1.21 (0.91-6.56)	2.83±0.82 (1.5-4.14)	3.30±1.46 (1.01-6.17)	3.27±1.52 (0.64-12.99)	0.725 (0.44)	
BAMS	31.22±4.17 (25.57-43.14)	29.77±1.29 (27.61-32.22)	30.07±2.47 (26.82-34.76)	30.27±3.15 (21.36-46.67)	0.467 (0.85)	
BADS	71.43±4.93 (57.68-76.99)	72.42±1.5 (70.55-75.33)	69.81±3.54 (63.11-75.71)	71.2±4.00 (54.04-84.43)	0.438 (0.91)	
BA	79.87±3.41 (74.26-86.9)	79.81±1.87 (77-82.76)	79.95±2.5 (76.34-84.86)	79.88±2.61 (70.74-89.68)	0.999 (0.01)	
TRIA	16.05±2.4 (10.08-19.7)	16.36±1.42 (13.79-18.31)	16.47±2.04 (12.28-20.26)	16.35±2.05 (8.79-24.34)	0.899 (0.20)	
TA	4.08±1.38 (2.31-8.20)	3.83±0.65 (2.94-4.79)	3.58±0.77 (2.38-5.02)	3.77±0.82 (1.54-7.38)	0.225 (1.46)	
C-FUC	21.64±4.53 (12.13-31.5)	21.47±3.32 (16.4-26.76)	24.61±5.45 (18.06-39.68)	22.71±4.38 (10.43-36.42)	0.167 (1.69)	
A-FUC	3.50±1.24 (1.79-6.51)	3.05±0.68 (2.18-4.04)	3.11±0.62 (2.14-4.59)	3.11±0.98 (1.43-11.05)	0.261 (1.34)	
A2	0.19±0.07 (0.12-0.40)	0.20±0.07 (0.1-0.3)	0.13±0.06 (0.07-0.25)	0.18±0.11 (0.04-2.01)	0.345 (1.11)	
G0	4.48±1.20 (2.23-7.38)	4.67±1.18 (3.41-6.76)	3.01±0.81 (1.77-4.83)	4.07±1.36 (1.39-12.84)	0.004 (4.42)	ce 0.017; eU 0.023; UB 0.005
G1	9.16±1.34 (6.45-11.67)	9.94±1.19 (8.18-12.36)	9.04±1.23 (7.19-11.39)	9.29±1.48 (5.00-19.19)	0.493 (0.80)	
G2	66.23±3.73 (58.56-78.22)	65.2±2.03 (62.27-69.33)	67.89±2.25 (64.06-71.33)	66.53±2.74 (56.43-79.18)	0.110 (2.01)	
G3	13.42±2.54 (8.49-17.91)	13.21±1.21 (10.84-14.67)	13.62±2.32 (9.64-17.8)	13.54±2.18 (6.24-22.54)	0.960 (0.10)	
G4	1.28±0.54 (0.41-2.69)	1.33±0.45 (0.91-2.38)	1.49±0.52 (0.52-2.53)	1.41±0.48 (0.32-3.29)	0.461 (0.86)	
IgG glycans						
IgG_GP1	0.22±0.21 (0.05-1.06)	0.32±0.25 (0.08-0.75)	0.19±0.14 (0.05-0.55)	0.20±0.17 (0.02-1.55)	0.170 (1.68)	
IgG_GP2	0.96±0.32 (0.40-1.86)	1.16±0.71 (0.37-2.65)	0.60±0.25 (0.21-1.21)	0.88±0.58 (0.14-9.39)	0.060 (2.47)	
IgG_GP4	25.22±5.54 (13.66-39.43)	22.52±4.02 (18.57-29.68)	16.19±4.88 (6.93-23.3)	20.99±6.14 (6.48-47.89)	<0.001 (9.69)	ce 0.004; cB <0.001; eB <0.001
IgG_GP3	0.12±0.02 (0.08-0.15)	0.11±0.05 (0.04-0.18)	0.08±0.01 (0.07-0.09)	0.10±0.03 (0.03-0.18)	0.049 (3.05)	
IgG_GP5	0.24±0.10 (0.10-0.56)	0.33±0.16 (0.15-0.69)	0.29±0.19 (0.08-0.92)	0.28±0.14 (0.07-1.64)	0.297 (1.23)	
IgG_GP6	6.58±1.60 (4.6-12.08)	6.37±1.35 (4.78-8.93)	4.43±1.10 (2.42-6.17)	5.61±1.63 (2.1-12.86)	<0.001 (8.17)	ce 0.010; cB 0.003; eU 0.019; eB <0.001
IgG_GP7	0.70±0.36 (0.25-1.61)	0.9±0.47 (0.32-1.78)	0.59±0.28 (0.27-1.29)	0.72±0.42 (0.11-4.06)	0.323 (1.16)	
IgG_GP8	16.69±1.99 (13.57-20.33)	15.84±2.17 (12.26-18.37)	17.27±2.87 (11.4-22.98)	16.65±2.21 (0.52-22.65)	0.446 (0.89)	
IgG_GP9	8.22±1.04 (6.28-10.44)	8.08±1.19 (5.67-9.18)	8.36±1.65 (3.84-11.88)	8.16±1.4 (4.27-24.91)	0.923 (0.16)	
IgG_GP10	4.86±1.06 (2.96-7.59)	5.05±0.79 (4.16-6.57)	4.88±0.78 (3.73-6.48)	4.77±0.96 (2.57-8.63)	0.721 (0.45)	
IgG_GP11	0.75±0.13 (0.42-1.01)	0.88±0.17 (0.64-1.1)	0.74±0.19 (0.42-1.11)	0.75±0.17 (0.29-1.82)	0.159 (1.73)	
IgG_GP12	0.91±0.46 (0.22-2.15)	1.06±0.46 (0.54-1.95)	1.10±0.51 (0.49-2.36)	1.06±0.57 (0.10-3.91)	0.487 (0.81)	
IgG_GP13	0.26±0.17 (0.13-0.98)	0.3±0.11 (0.19-0.55)	0.26±0.08 (0.12-0.41)	0.26±0.13 (0.08-1.59)	0.807 (0.33)	
IgG_GP14	9.75±2.51 (4.81-17.54)	9.3±2.29 (6.84-13.72)	14.59±3.45 (8.74-20.94)	11.6±3.67 (3.39-25.82)	<0.001 (8.50)	ce 0.002; cB 0.018; eU 0.002; eB <0.001
IgG_GP15	1.41±0.28 (0.76-2.31)	1.5±0.23 (1.14-1.8)	1.76±0.38 (1.14-2.49)	1.55±0.36 (0.75-3.54)	0.009 (3.85)	eB 0.005
IgG_GP16	3.21±0.52 (2.35-4.88)	3.29±0.25 (2.88-3.62)	3.26±0.36 (2.55-3.89)	3.16±0.50 (1.71-4.82)	0.644 (0.56)	
IgG_GP17	2.32±1.47 (0.67-7.21)	2.98±1.68 (1.44-5.88)	2.55±1.54 (0.59-5.80)	2.79±1.83 (0.46-13.61)	0.444 (0.89)	
IgG_GP18	7.69±1.63 (4.39-12.05)	7.2±1.51 (5.05-9.86)	11.27±2.65 (6.47-17.31)	9.07±2.53 (3.29-19.38)	<0.001 (10.06)	ce 0.001; cB 0.008; eU <0.001; eB <0.001
IgG_GP19	2.20±0.60 (1.44-3.81)	2.34±0.37 (2.06-3.22)	2.32±0.61 (1.50-3.92)	2.35±0.60 (1.12-9.03)	0.535 (0.73)	
IgG_GP20	0.53±0.28 (0.14-1.13)	0.89±0.66 (0.29-2.07)	0.61±0.51 (0.14-2.26)	0.62±0.45 (0.08-3.46)	0.199 (1.55)	
IgG_GP21	2.72±1.86 (0.45-7.40)	4.93±3.54 (1.95-12)	3.67±3.57 (0.48-15.60)	3.52±2.78 (0.39-22.46)	0.153 (1.76)	

IgG_GP22	0.30±0.16 (0.08-0.78)	0.37±0.13 (0.22-0.61)	0.30±0.23 (0.06-1.17)	0.32±0.17 (0.04-1.46)	0.600 (0.62)
IgG_GP23	1.83±0.57 (0.86-3.43)	1.79±0.34 (1.18-2.23)	2.25±0.50 (1.31-3.32)	2.07±0.63 (0.70-4.67)	0.040 (2.79)
IgG_GP24	2.41±0.84 (1.23-5.44)	2.6±0.5 (2.12-3.81)	2.53±0.63 (1.25-3.60)	2.59±0.79 (1.08-12.45)	0.609 (0.61)

pair-wise comparisons across groups, where B denotes bilateral group, U unilateral, e early-onset and c controls.
